# Remitting seronegative symmetrical synovitis with pitting edema syndrome induced by pembrolizumab in patient with urothelial carcinoma

**DOI:** 10.1002/iju5.12426

**Published:** 2022-05-03

**Authors:** Akihiro Yoshimura, Kazuaki Yamanaka, Rei Tadokoro, Teppei Wakita, Shota Fukae, Takahiro Yoshida, Masahiro Sekiguchi, Hidefumi Kishikawa

**Affiliations:** ^1^ Department of Urology Hyogo Prefectural Nishinomiya Hospital Nishinomiya City Hyogo Japan; ^2^ Department of Rheumatology Hyogo Prefectural Nishinomiya Hospital Nishinomiya City Hyogo Japan; ^3^ Present address: Department of Urology Graduate School of Medicine Faculty of Medicine Osaka University Yamadaoka 2‐15 Suita City Osaka 565‐0871 Japan

**Keywords:** connective tissue disease, drug related side effects and adverse reactions, immune checkpoint inhibitors, pembrolizumab, transitional cell carcinoma

## Abstract

**Introduction:**

Recent introduction of immuno‐oncology drugs such as pembrolizumab has resulted in improved outcomes for urothelial carcinoma patients. However, immune‐related adverse events generally show great variance and are often difficult to diagnose and control.

**Case presentation:**

An 84‐year‐old Japanese male with urothelial carcinoma metastasis to the lungs after a laparoscopic left radical nephroureterectomy procedure was treated with pembrolizumab, an immuno‐oncology drug, as second‐line therapy. At week 6, inflammatory arthralgia involving the hands and shoulder joints, and edema of the hands were presented. The diagnosis was remitting seronegative symmetrical synovitis with pitting edema syndrome. Pembrolizumab was discontinued, and oral corticosteroid therapy was started. Two months later, pembrolizumab treatment was resumed because of a significant improvement in patient condition.

**Conclusion:**

Although rare, immune‐related adverse events are occasionally encountered during the use of immune‐oncology drugs; thus, early diagnosis and appropriate treatment are important.

**Abbreviations & Acronyms**
CT: computed tomography
I‐O: immuno‐oncology
irAE: immune‐related adverse event
RS3PE: remitting seronegative symmetrical synovitis with pitting edema

**Keynote message**

It is important to note that pembrolizumab, a drug prescribed for the treatment of urothelial carcinoma, activates the immune system and can cause RS3PE syndrome, a relatively rare collagen disease, as an immune‐related adverse event.

## Introduction

Recent introduction of I‐O drugs such as pembrolizumab have resulted in improved outcomes for urothelial carcinoma patients. However, as compared to cases undergoing conventional chemotherapy, irAEs have greater variance and are often more difficult to diagnose and control. For example, while arthritis is a common symptom of rheumatic irAE, there are few reports of occurrence of RS3PE syndrome as an irAE. This syndrome, first reported in 1985, is an atypical presentation of arthritis, characterized by sudden onset symmetric distal synovitis and pitting edema of the hands and occurs more often in older individuals with a male predominance.^1^ Inflammatory markers such as CRP are usually elevated, while the rheumatoid factor is negative, and this syndrome is usually highly sensitive to low‐dose corticosteroid therapy.^2^ We report a case of RS3PE syndrome in a metastatic urothelial carcinoma patient that was presented during pembrolizumab therapy.

## Case presentation

**Fig. 1:**
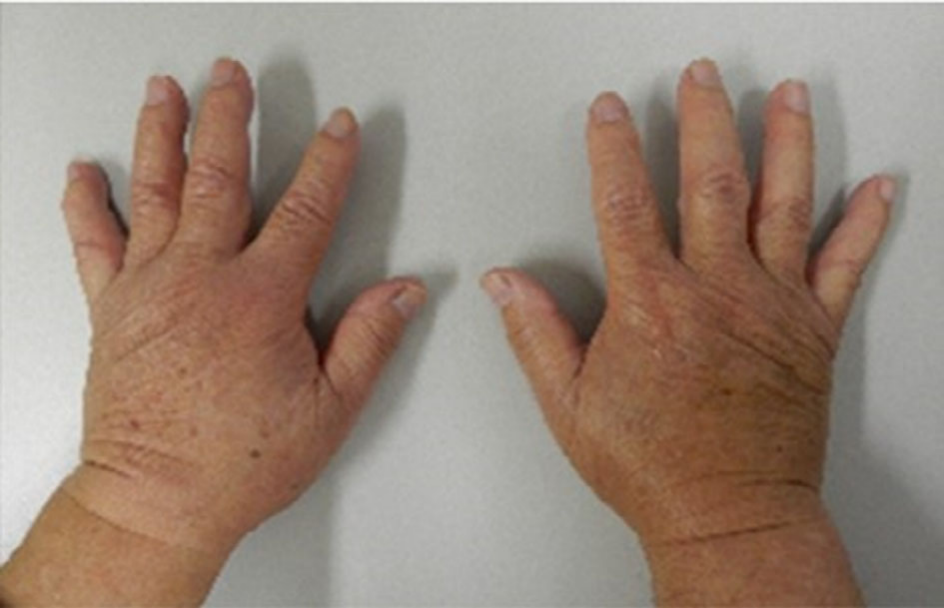
Pitting edema of hands in patient with arthralgia.

An 84‐year‐old Japanese male was presented with left abdominal pain, and CT scan findings revealed a tumor of the left ureter and hydronephrosis. The patient underwent a laparoscopic left radical nephroureterectomy, and a pathological examination indicated urothelial carcinoma with invasion of periureteric fat and metastasis of lymph nodes in the renal hilum. Four months later, a chest CT scan showed multiple lung metastases, and chemotherapy (gemcitabine and carboplatin) was then given as first‐line therapy. After 10 courses, the areas of lung metastasis were increased and the treatment was changed to pembrolizumab (200 mg, every 3 weeks). Following the second cycle of pembrolizumab infusion, acute pain and swelling in the shoulders, elbows, wrists, and finger joints developed, accompanied by edema in both hands (Fig. 1). Laboratory tests revealed elevated inflammatory markers (CRP, 7.61 mg/dL; matrix metalloproteinase‐3, 330.1 ng/mL), whereas the rheumatoid factor and anti‐cyclic citrullinated peptide antibody were negative. Ultrasonography showed increased vascularization in symptomatic joints, while X‐ray analysis of the hands did not show any osteoclastic lesion. Based on the symptoms and laboratory findings, the diagnosis was the RS3PE syndrome. The medical history of the patient included early‐stage gastric cancer and thyroid cancer, which had been treated surgically and were in remission. There was no past medical or family history of connective tissue disease or inflammatory arthritis.

Pembrolizumab treatment was suspended and prednisolone, an oral corticosteroid, was started at 0.25 mg/kg/day (15 mg/day). The next day, joint pain began to improve quickly, while the other symptoms showed gradual improvement. After 2 weeks of treatment, all symptoms had disappeared and inflammatory markers returned to normal. Thus, pembrolizumab therapy was resumed and prednisolone gradually tapered to 9 mg/day at 2 months after beginning administration. After 6 months of pembrolizumab retreatment, prednisolone was tapered to 3 mg/day. The lung metastasis did not show progression during the cessation of pembrolizumab or after its beginning, and the patient has remained stable after resumption without RS3PE syndrome flare‐up or other irAEs. The present strategy for this case is further reduction of prednisolone medication and eventual termination in the absence of a flare‐up of symptoms.

## Discussion

I‐O drugs exert antitumor effects by activating immune response against tumor cells, though it has also been suggested that they cause irAEs due to excessive autoimmune response. RS3PE syndrome is an uncommon irAE, with only eight known cases reported at the time of writing (Table 1). Those show that patients with RS3PE syndrome respond markedly to steroid therapy and most go into remission; thus, progressive reduction or discontinuation of the steroid can be expected. As for RS3PE syndrome that develops during I‐O drug administration, the majority of reports suggest discontinuation of any such drug as a treatment strategy. However, some have indicated that the clinical condition of patients affected by this syndrome could be controlled with an I‐O drug; thus, the need for discontinuation remains inconclusive. Furthermore, reports of flare‐ups of irAEs after restarting I‐O drug treatment and during steroid reduction have been presented; thus, careful monitoring of symptoms is required even after resumption.^3^, ^4^

**Table 1:** Present and previous reports of cases of RS3PE syndrome (except for paraneoplastic RS3PE syndrome) that developed during I‐O drug therapy

Reference (no.)	Age (years)	Gender	Disease	I‐O drug	Onset of symptoms after initial I‐O drug administration	Initial treatment for RS3PE	Discontinuation of I‐O drug	Therapeutic course	Readministration of I‐O drug	Symptom relapse after readministration
Kim *et al*.^10^	59	Male	Prostate cancer	Ipilimumab + apalutamide + abiraterone acetate	3 courses (every 3 weeks)	Weekly methotrexate (15 mg) with daily folic acid (1 mg)	Yes	Remission	No	
Wada *et al*.^11^	70	Male	Malignant melanoma	Nivolumab	7 courses (every 2 weeks)	Prednisolone (10 mg/day)	No	Remission	Yes	Yes ( symptoms relapsed after each administration of nivolumab and could be controlled by small amount of steroid)
Hansmaennel *et al*.^12^	79	Male	Malignant melanoma	Pembrolizumab	11 months	Prednisone (60 mg/day )	No	Remission	Yes	Unknown
Gauci *et al*.^13^	80	Male	Malignant melanoma	Nivolumab	2 courses (every 2 weeks)	Corticosteroid (0.5 mg/kg/day)	Yes	Remission	Yes	Yes (spontaneous remission within 2 weeks without increase in corticosteroid dose)
Ngo *et al*.^14^	70	Male	Malignant melanoma	Nivolumab	4 months	Prednisone (0.5 mg/kg/day)	No	Remission and relapse after tapering corticosteroid	Yes	No (prednisone maintained at 7.5 mg/day)
Filetti *et al*.^15^	57	Female	Lung cancer	Nivolumab	18 months (28 nivolumab administrations)	Prednisone (1 mg/kg/day)	Yes	Remission	Yes	No
Amini‐Adle *et al*.^16^	70	Male	Malignant melanoma	Nivolumab + ipilimumab	6 weeks	Prednisone (1000 mg/day)	Yes	Remission and relapse after tapering corticosteroid	No	
Redman *et al*.^17^	70	Male	Prostate cancer	Durvalumab	1 week	Prednisone (15 mg/day)	Yes	Remission	Yes	Yes (symptoms relapsed after steroid withdrawal)
Present patient	84	Male	Urothelial carcinoma	Pembrolizumab	2 courses (every 3 weeks)	Prednisolone (30 mg/day)	Yes	Remission	Yes	No

RS3PE syndrome has been regarded as a paraneoplastic syndrome in 24.7% of all reported cases.^5^ When this syndrome develops in patients with urothelial carcinoma being treated with an immune checkpoint inhibitor, it is necessary to distinguish between paraneoplastic syndrome and an irAE. However, it is difficult to differentiate between these etiologies as there are no apparent differences in clinical symptoms, laboratory findings, or image examination findings. Poor response to corticosteroid treatment has been observed in paraneoplastic RS3PE syndrome patients.^6^ Yamamoto *et al*. also reported RS3PE syndrome that developed after 17 courses of pembrolizumab therapy for urothelial carcinoma, in which improvement was noted with low‐dose corticosteroid treatment and continuation of pembrolizumab.^7^ However, findings presented in that study did not elucidate whether irAE or paraneoplastic syndrome was the cause. Symptoms in the present case were noted early following the start of pembrolizumab administration, with recovery demonstrated after temporarily stopping the I‐O drug and treatment with low‐dose prednisolone. Cancer progression was stable; thus, we considered that RS3PE syndrome was caused by an irAE. Accumulation of case reports is necessary to elucidate the pathogenesis of RS3PE syndrome in association with irAE and paraneoplastic disease in order to select appropriate treatment.

Some reports have found that low‐dose steroid therapy does not alter the anti‐tumor effects of I‐O drugs, while others have noted that steroids reduced the effect of I‐O drugs, leading to a poor cancer prognosis.^8^, ^9^ The risks and benefits of using steroid treatment in patients receiving immune checkpoint inhibitor therapy should be carefully considered.

## Conclusion

We report here a case of RS3PE syndrome that developed during pembrolizumab therapy for metastatic urothelial carcinoma. The symptoms were significantly improved by temporary discontinuation of the drug and corticosteroid therapy.

## Author Contributions

Akihiro Yoshimura: Data curation; Investigation; Visualization; Writing – original draft. Kazuaki Yamanaka: Conceptualization; Project administration; Writing – review & editing. Rei Tadokoro: Writing – review & editing. Teppei Wakita: Writing – review & editing. Shota Fukae: Writing – review & editing. Takahiro Yoshida: Writing – review & editing. Masahiro Sekiguchi: Writing – review & editing. Hidefumi Kishikawa: Writing – review & editing.

## Conflict of interest

The authors declare no conflict of interest.

## Approval of the research protocol by an Institutional Reviewer Board

Not applicable.

## Informed consent

Informed consent was obtained from the patient for publication of the case details.

## Registry and the Registration No. of the study/trial

Not applicable.
